# Transcriptomic analysis of the response of *Acropora millepora* to hypo-osmotic stress provides insights into DMSP biosynthesis by corals

**DOI:** 10.1186/s12864-017-3959-0

**Published:** 2017-08-14

**Authors:** Catalina Aguilar, Jean-Baptiste Raina, Cherie A. Motti, Sylvain Fôret, David C. Hayward, Bruno Lapeyre, David G. Bourne, David J. Miller

**Affiliations:** 1 AIMS@JCU, and Department of Molecular and Cell Biology, James Cook University, Townsville, 4811 Queensland Australia; 2 ARC Centre of Excellence for Coral Reef Studies, James Cook University, Townsville, 4811 Queensland Australia; 3 Climate Change Cluster (C3), Faculty of Science, University of Technology, Sydney, NSW 2007 Australia; 4 Australian Institute of Marine Science, Townsville, 4810 Queensland Australia; 5 Evolution and Ecology, Research School of Biology, The Australian National University, Canberra, ACT 2601 Australia; 6 Laboratoire d’excellence CORAIL, Centre de Recherches Insulaires et Observatoire de l’Environnement (CRIOBE), Moorea, B.P. 1013 Papeete, French Polynesia; 7 College of Science and Engineering, James Cook University, Townsville, 4811 Queensland Australia

**Keywords:** DMSP pathway, *Acropora millepora*, Coral, Salinity stress, Methionine

## Abstract

**Background:**

Dimethylsulfoniopropionate (DMSP) is a small sulphur compound which is produced in prodigious amounts in the oceans and plays a pivotal role in the marine sulfur cycle. Until recently, DMSP was believed to be synthesized exclusively by photosynthetic organisms; however we now know that corals and specific bacteria can also produce this compound. Corals are major sources of DMSP, but the molecular basis for its biosynthesis is unknown in these organisms.

**Results:**

Here we used salinity stress, which is known to trigger DMSP production in other organisms, in conjunction with transcriptomics to identify coral genes likely to be involved in DMSP biosynthesis. We focused specifically on both adults and juveniles of the coral *Acropora millepora*: after 24 h of exposure to hyposaline conditions, DMSP concentrations increased significantly by 2.6 fold in adult corals and 1.2 fold in juveniles*.* Concomitantly, candidate genes enabling each of the necessary steps leading to DMSP production were up-regulated.

**Conclusions:**

The data presented strongly suggest that corals use an algal-like pathway to generate DMSP from methionine, and are able to rapidly change expression of the corresponding genes in response to environmental stress. However, our data also indicate that DMSP is unlikely to function primarily as an osmolyte in corals, instead potentially serving as a scavenger of ROS and as a molecular sink for excess methionine produced as a consequence of proteolysis and osmolyte catabolism in corals under hypo-osmotic conditions.

**Electronic supplementary material:**

The online version of this article (doi:10.1186/s12864-017-3959-0) contains supplementary material, which is available to authorized users.

## Background

Dimethylsulphoniopropionate (DMSP) and its volatile breakdown product dimethylsulphide (DMS) are key components in the global sulphur cycle; the conversion of DMSP to DMS delivers biogenic sulphate aerosols into the marine boundary layer, thereby transferring sulphur from the oceans to the atmosphere [1]. DMS can subsequently be oxidized into sulphate particles and, when combined with ultrafine sea salt and other marine organic aerosols, contributes to the formation of clouds, increasing their reflectance and thereby acting in local climate regulation [2]. DMSP is produced by several classes of algae and a few higher plants [3, 4]. In addition, coral reefs are hotspots for the production of this compound [5, 6]. This high production of DMSP has previously been ascribed solely to the high densities of the dinoflagellate *Symbiodinium* present in coral tissues. It is becoming increasingly clear, however, that photosynthesis is not a prerequisite for DMSP production: the coral animal [7] and some heterotrophic bacteria [8] have recently been shown to produce DMSP. However, the molecular mechanisms underlying the production of DMSP by corals are unknown and are only partially understood in other eukaryotes.

**Fig. 1:**
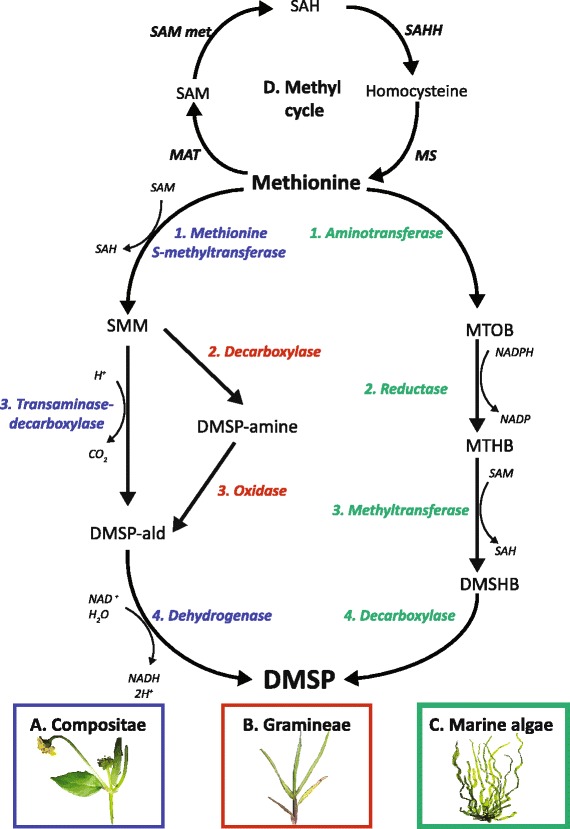
Pathways of DMSP biosynthesis in higher plants and marine algae (adapted from [4]). **a** Compositae pathway (described in *Wollastonia biflora*, in *blue*). **b** Gramineae pathway (described in *Spartina alterniflora*, in *blue/red*). **c** Marine algal pathway (described in *Ulva intestinalis*, in *green*). **d** Methyl cycle and the enzymes involved in methionine biosynthesis. Dimethylsulphonio-2-hydroxybutyrate (DMSHB); dimethylsulphoniopropionate (DMSP); DMSP-aldehyde (DMSP-ald); 4-methylthio-2-hydroxybutyrate (MTHB); 2-oxo-4-methylthiobutanoate (MTOB); *S*-adenosylhomocysteine (SAH); *S*-adenosylmethione (SAM); *S*-methylmethionine (SMM). Enzyme types and associated cofactors are shown in italics (refer to Table 1 for the enzyme names)

DMSP biosynthesis is thought to have evolved independently at least three times; two different pathways have been described in higher plants [9, 10], and the third was identified in the marine macroalga *Ulva intestinales* [11] but might also operate in several phytoplankton species and heterotrophic bacteria (Fig. 1). The common denominator in these three pathways is the use of the sulphur-containing amino acid methionine as a precursor. The identities of the intermediates involved in each pathway have largely been established, providing insights into the classes of enzymes involved. However, with the exception of *dsyB* which was recently identified in Alphaproteobacteria [8], the identities of the genes involved are unknown at this time. Candidate genes for the algal pathway have emerged from proteomic and gene expression analyses under conditions that lead to increased DMSP production. Proteomic analyses of DMSP-producing diatoms implicated specific aminotransferases, reductases, methyltransferases and decarboxylases [12, 13] on the basis of their increased abundance under hypersaline conditions, though their involvement in DMSP synthesis remains to be confirmed.

A range of cellular functions have been attributed to DMSP: it can act as an osmolyte [14] or cryoprotectant [15, 16]. DMSP and its breakdown products acrylate, DMS and dimethylsulfoxide (DMSO) also possess antioxidant capabilities, and are capable of scavenging hydroxyl radicals and reactive oxygen species (ROS), suggesting potential functions in the stress responses of organisms such as corals [17]. Consistent with this, oxidative stress triggered by decreased salinity resulted in increased conversion of DMSP to DMSO in the reef-building coral *Acropora millepora* [18]. However this effect was not observed with other coral species, such as *Stylophora pistillata* and *Pocillopora damicornis* [18]. Thus, whilst DMSP has been implicated in ROS-scavenging in at least some corals, an osmoregulatory role remains an additional possibility.

Although corals have traditionally been thought of as stenohaline osmo-conformers [19], shallow water corals can experience major fluctuations in salinity and must therefore have mechanisms to tolerate these environmental conditions. Currently limited data are available on the effects of hyperosmotic stress on corals, but there is evidence that corals can tolerate acute exposure to hypersaline (40 practical salinity units (PSU)) conditions [20]. Moreover, coral reefs occur in the Arabian Gulf and Gulf of Oman at 40–42 PSU, and appear to be adapted to these conditions [21]. On the Great Barrier Reef (GBR), rain associated with tropical cyclones can lower the salinity of surface waters significantly (up to 7–10 PSU) [22], with these hyposaline conditions sometimes prevailing for weeks [23]. Hyposaline conditions can lead to coral mortality and changes in coral community composition; however, the response seems to vary among species and through time [24]. Heavy rainfall, induced by the increased occurrence and intensity of tropical storms and cyclones [25], is likely to expose coral reefs to more extreme and sudden salinity variations.

The genome of the reef-building coral *Acropora millepora* encodes orthologs of the reductase and methyltransferase (Fig. 1c, steps 2 and 3) implicated in DMSP biosynthesis in algae, suggesting that corals also use an algal-like pathway to produce DMSP from methionine [7]. To better understand the role and route of DMSP production in corals, the transcriptomic response of *A. millepora* to salinity stress was investigated. Assuming that DMSP acts as an osmolyte in corals, we hypothesized that genes involved in its synthesis will be up-regulated under salinity stress. Adult colonies (harboring DMSP-producing photosynthetic symbionts), as well as aposymbiotic juveniles (devoid of any photo-symbionts) of *A. millepora* were exposed to hyposaline conditions reflecting those experienced in extreme weather events (25 PSU for the adults and 28 PSU for the juveniles) in parallel experiments and hypersaline (40 PSU) conditions for the adults. The analyses presented here focused on candidate genes encoding enzyme classes that could fulfill each of the steps necessary to transform methionine into DMSP. The expression data support the idea that corals are equipped with the necessary enzymatic machinery for DMSP biosynthesis and can rapidly change the expression of the corresponding genes.

## Methods

### Adult salinity stress experiment

The work described here was carried out under GBRMPA permit G09/30327.1. Eight *A. millepora* colonies were collected from Orpheus Island, Queensland, Australia (18°39′52. 43″S, 146°29′42.38″E) in June 2013 and transferred to the Australian Institute of Marine Science’s National Sea Simulator (SeaSim) facilities where the colonies were acclimated for 14 days in outdoor aquaria at ~27 °C. Each colony was fragmented into 25 nubbins (~6 cm) that were randomly distributed across three 50 l tanks. The tanks were linked to a computer controlled flow-through system supplying 0.04 μm filtered seawater (FSW) maintained at 25.7 °C (±0.6 °C) and an ambient salinity of 35 PSU. UV-filtered lights were mounted above each tank and nubbins were exposed to an intensity of 250 μE over a 12:12 h light/dark cycle (type of lights: 400 W metal halide lamps, BLV). The nubbins were acclimated in this system for a further 19 days to allow recovery. At the beginning of the experiment, the flow was stopped to ensure no water exchange (tanks were oxygenated via a pump) (Tunze 6015). The nubbins were subsequently exposed to one of three salinity regimes for 24 h: ambient/control salinity of 35 PSU (*n* = 72) for the duration of the experiment, low salinity of 25 PSU (*n* = 62) or high salinity of 40 PSU (*n* = 62). Some nubbins were used as test samples before the start of the experiment, hence the larger number of nubbins in the control treatment. These salinity regimes were chosen based on realistic fluctuations experienced by corals in the field. The 25 PSU FSW was prepared by diluting 700 ml of 35 PSU FSW with 300 ml reverse-osmosis water while the 40 PSU FSW was prepared by adding 11 g of Red Sea Coral Pro Salt (Red Sea Aquatics Ltd., Houston, TX) to 1 l of 35 PSU FSW. The temperature during the treatment period was maintained at 25.9 °C (±0.7). Salinity was monitored using a water quality meter (TPS 90FL, ThermoFisher).

Coral nubbins (*n* = 2 per colony) were sampled at three time points for RNA analysis, and quantitative nuclear magnetic resonance (qNMR) analysis: prior to the salinity change, and after 1 and 24 h post the salinity change. Nubbins for RNA analysis were snap frozen in liquid nitrogen and stored at −80 °C, whereas nubbins for qNMR analysis were immediately extracted in 5 ml of HPLC-grade methanol (details provided below). Another set of nubbins (*n* = 1 per colony) were collected for the determination of *Symbiodinium* density at four time points (prior salinity change, 1 h, 12 h and 24 h).

### *Symbiodinium* photosystem II photochemical efficiency, density estimation and genotyping

A diving pulse amplitude modulated (PAM) (Walz Gmbh, Germany) fluorometer was used to measure the photosystem II (PSII) photochemical efficiency of *Symbiodinium* associated with the adult coral nubbins. Measurements were taken 1 day before, and 8, 16, 28 h after changing the salinity, by taking 3 replicates per 23 nubbins in each condition. *Symbiodinium* density estimation was conducted as described in Raina et al. [7]; for each homogeneous extract, 6 replicate measurements were recorded at 600 nm on a DSM-Micro densitometer (Laxco, Washington). For genotyping, DNA was extracted from the crushed coral (see RNA extraction) using SNET buffer (20 mM Tris-HCl pH 8.0, 5 mM EDTA, 1% SDS (*w*/*v*), 400 mM NaCl, 400 μg ml^−1^ Proteinase K) and incubated overnight at 55 **°**C. The supernatant was transferred to an equal volume of phenol-chloroform mixture (1:1) and precipitated with isopropanol. The DNA pellet was solubilized in ~50 μl of sterile water and stored at −20 **°**C. The *Symbiodinium* type was determined by ITS sequencing using the primers “ITSintfor2” (5’GAATTGCAGAACTCCGTG-3′) and “ITS2CLAMP” (5’GGGATCCATATGCTTAAGTTCAGCGGGT-3′) [26]. All *A. millepora* colonies harboured *Symbiodinium* clade C1.

### Juvenile salinity stress experiment

*A. millepora* colonies were collected from Trunk Reef, GBR, Australia (18°22′15.10″S/146°48′27.82″E) and transferred to the SeaSim facilities at the Australian Institute of Marine Science prior to the predicted spawning in November 2013. Colonies were individually placed in 70 l tanks with 0.2 μm FSW. Coral gametes fertilization and embryos treatment methods were as described in Tebben et al. [27] and Raina et al. [7]. After 13 days, *Symbiodinium*-free coral larvae were collected using 1 μm mesh and washed carefully three times in 0.2 μm FSW to remove loosely attached material including potential DMSP-degrading bacteria. A coral settlement cue (5 μl) derived from crustose coralline algae extract (CCA; see Tebben et al. [28], the cue did not contain DMSP) was added to 6-well plates and allowed to evaporate completely. Subsequently, 40 ml of 35 PSU FSW was added to each well and gently mixed. Competent, washed coral larvae (*n* = 40) were then introduced carefully into each well and the plates maintained in the dark at 26.3 °C (±0.005) to prevent growth of photosynthetic organisms. Throughout the incubation phase, the FSW (35 PSU) was replaced on alternate days. Four days post-settlement (T_0_), plates were separated into two groups: 16 plates were maintained at 35 PSU (control salinity) while the water in the remaining 16 plates was exchanged with 28 PSU water (salinity stress treatment). The 28 PSU FSW was prepared by diluting 800 ml of 35 PSU FSW with 250 ml reverse-osmosis water. During the treatment period the water was exchanged (maintaining the PSU) after 12 h and 24 h to ensure adequate oxygenation. Samples were collected at T_0,_ 24 (T_24_), and 48 h (T_48_)_,_ for RNA and qNMR analysis. The juveniles were incubated longer than the adults based on results derived from a pilot study. The size of each settled juvenile in the sampled well was measured using a motorized stereomicroscope (Leica Microsystems MZ16A) operating with the Application Suite Version 3.8 software. The average juvenile size at 48 h was 1.27 mm^2^ (±0.06).

### DMSP quantification by qNMR analysis

DMSP and acrylate in adult nubbins and settled juveniles were quantified according to Raina et al. [7] with minor modifications. Briefly, coral nubbins were extracted in methanol for 30 min with sonication followed by a second extraction with an additional 2 ml of methanol for 10 min, after which the extracts were pooled and analysed via ^1^H NMR as in Raina et al. [7] using the ERETIC method [29]. The surface area of each individual adult nubbin was used to normalise the corresponding qNMR and *Symbiodinium* density data. Nubbins were bleached (10% bleach) and then lyophilized (Dynavac Freeze Drier FD12) with the surface area determined using the wax dipping technique originally described by Veal et al. [30].

For juveniles, seawater was decanted from individual wells and residual seawater gently absorbed using a sterile cotton tip, taking care not to disturb the animal. CD_3_OD (300 μl) and D_2_O (200 μl) were added to each well. Plates were gently shaken for 30 s and a 200 μl aliquot transferred into a 3 mm Bruker MATCH NMR tube for immediate analysis. In addition, negative control wells containing no larvae or settled juveniles, but which did contain the CCA-derived settlement cue, were extracted following the same procedure. The concentrations of DMSP and acrylate were normalized initially to the number of settled coral juveniles in the respective well. They were then normalized to the averaged surface area of the juveniles as in Raina et al. [7].

DMSP concentration data were analysed using the open source software R Version 3.1.0 (R Core team, 2014) using the “car” [31] and “doBy” [32] libraries. Multivariate analyses of variance MANOVA were used to test for changes in DMSP concentration over the course of the experiment. Repeated measures ANOVA were used to test for difference in DMSP concentration at each time point and over time (Additional file 1: Table S1).

### RNA extraction sequencing and gene expression analyses

Adult nubbins from the 25 and 35 PSU treatments were crushed in liquid nitrogen and ~1 g of the resulting powder homogenized for 15 min by vortexing in 3 ml of TRIzol Reagent (Invitrogen), followed by centrifugation at 4000 g for 15 min. The supernatant was recovered with a 1 ml pipet leaving the coral tissue pellet. 4-Bromo-2-chlorophenol (150 μl) was added to the recovered supernatant according to the TRIzol manufacturer’s specifications with a slight modification, 0.5 ml of 100% isopropanol was replaced with a mixture of 300 μl 100% isopropanol and 200 μl of high-salt buffer (0.8 M Na citrate, 1.2 M NaCl) per 1.5 ml of TRIzol in the precipitation step. The RNA pellet was solubilized in ~50 μl of RNAse-free water and stored at −80 **°**C.

Coral juveniles were sampled by removing the water and adding 1.5 ml of RNA*later* (Ambion, cat# AM7021) simultaneously to each well and scraping the content with a sterile 200 μl plastic tip to transfer the contents into a 2 ml tube and stored at −20 **°**C. Total RNA of the 24 juvenile samples was extracted using the RNAaqueous-Micro total RNA isolation kit (AM1931, AMBION). The quality and quantity of RNA preparations were determined using a Bioanalyzer (Agilent 2100 Bioanalyzer) using samples prepared following the Agilent RNA 6000 Nano Kit instructions (cat # 7067–1511).

RNAseq libraries were constructed using the NEB Next Ultra Directional RNA Library Prep Kit for Illumina (NEB, E7420S) following the manufacturers recommended protocol and 100 bp paired-end sequence data obtained using a HiSeq 2000 at the Biomolecular Resource Facility (John Curtin School of Medical Research, Australian National University). Reads were mapped onto the *Acropora millepora* genome using TopHat2 [33] to produce a count data gene expression matrix for subsequent analysis. Counts were generated using htseq-count [34].

Data were analysed in DESeq2 package [35] in R (R Core Team 2014) using a design formula for differential gene expression that tests for the effects of salinity, controlling for the effect of the colony type and running the default functions for estimating size factors, dispersion and negative binomial Wald Test. Log_2_ fold changes (log_2_FC) in gene expression levels were obtained in DESeq2 by comparing control vs. salinity treatment in six different cases: (i) control vs. treatment at 1 h in the adults, (ii) control vs. treatment at 24 h in the adults, (iii) control vs. treatment at 1 and 24 h in the adults (iv) control vs. treatment at 24 h in the juveniles, (v) control vs. treatment at 48 h in the juveniles, and (vi) control vs. treatment at 24 and 48 h in the juveniles. False discovery rate (FDR) adjusted *p* values for each gene, was controlled at 5% according to the methods of Benjamini and Hochberg [36].

### Identification of candidate genes

To identify homologs of the known algal and plant DMSP biosynthesis enzymes in the coral genome, protein sequences from the diatom *Fragilariopsis cylindrus* v1.0 (algal pathway) [12, 13] in addition to sequences from the two known enzymes involved in the plant pathway (Enzyme Commission (EC) 2.1.1.12 and 1.2.1.3, downloaded from http://www.uniprot.org) were used to retrieve protein family (Pfam) domain and gene ontology (GO) annotation. In addition to complete sequences, protein domains were used to search the *A. millepora* genome for homologs of the algal and plant enzymes. Additionally, sequences with characteristic GO domains of the enzymes involved in DMSP biosynthesis from four algae and two plant genomes were retrieved and blasted against the *A. millepora* genome (E-value was set to 0.003, retrieving at least five sequences). Sequences were retrieved from: the marine microalga *Emiliania huxleyi* [37], the green alga *Chlamydomonas reinhardtii* v5.5 [38], the diatom *Thalassiosira pseudonana* v3.0 [13], the dinoflagellate *Symbiodinium minutum* Clade B1 v.1.0 [39] (dataset downloaded from http://marinegenomics.oist.jp/symb/viewer/info?project_id=21, last accessed October 27, 2014), and the flowering plants *Arabidopsis thaliania TAIR10* [40] and *Brachypodium distachyon v2.1* [41]. All the databases (except for the *S. minutum*) were downloaded from the U.S. Department of Energy Joint Genome Institute (JGI; http://genome.jgi-psf.org, last accessed October 15, 2014). The nomenclature of *A. millepora* proteins used here is based on BlastP searches of non-redundant protein sequences at NCBI or by hidden Markov models in HMMER (http://hmmer.janelia.org); [42]) assignments (results are listed in Table 2 and Additional file 2: Table S4). KEGG orthology (KO) identifiers were used to retrieve EC numbers and search for characteristics in the enzyme information system BRaunschweig ENzyme DAtabase (BRENDA; http://www.brenda-enzymes.org/index.php) and the metabolic pathways database (MetaCyc; http://metacyc.ai.sri.com). After obtaining the BlastP results based on the *A. millepora* gene predictions, differentially up-regulated genes (FDR < 0.05) in any of the datasets were subject to further analyses, and the sequences are provided as Supplementary data (Additional file 3).

**Table 1:** List enzyme abbreviations and corresponding EC numbers

Abbrev.	Enzyme name	EC number
BADH	Betaine-aldehyde dehydrogenase	1.2.1.8
BHMT	Betaine-homocysteine methyltransferase	2.1.1.5
CDH	Choline dehydrogenase	1.1.99.1
DMGDH	Dimethylglycine dehydrogenase	1.5.8.4
GNMT	Glycine N-methyltransferase	2.1.1.20
MAT	Methionine adenosyltransferase	2.5.1.6
MS	Methionine synthase	2.1.1.13
MTHFR	Methylenetetrahydrofolate reductase	1.5.1.20
SAHH	S-adenosylhomocysteinase	3.3.1.1
SAM met	S-adenosylmethione methyltransferase	2.1.1.37
SARDH	Sarcosine dehydrogenase	1.5.8.3
SHMT	Serine hydroxymethyltransferase	2.1.2.1

**Table 2:**
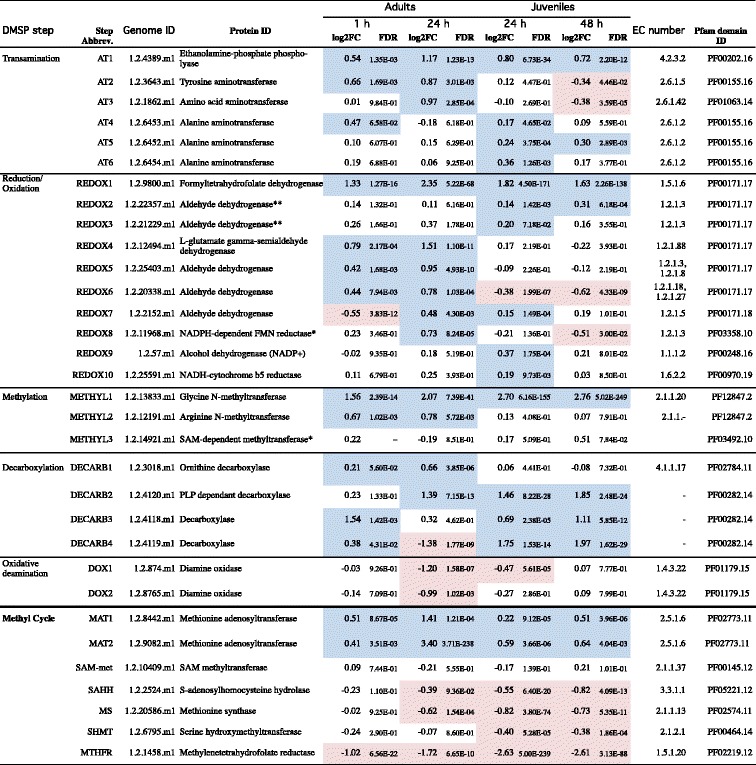
Changes in expression levels of candidate genes in *A. millepora* under hyposaline stress

For each candidate gene, the table provides log_2_ fold change (log_2_FC) and false discovery rate (FDR) data for the hyposaline treatment relative to the control. Blue shading indicates genes that were up-regulated; red shading indicates genes that were down-regulated (FDR <0.05)

*Candidates previously identified by Raina et al.*,* [7]

**Genes differentially up-regulated in the adult treatments when time was excluded as a factor (Additional file 2: Table S4)

## Results

### Concentration of DMSP in coral tissues

**Fig. 2:**
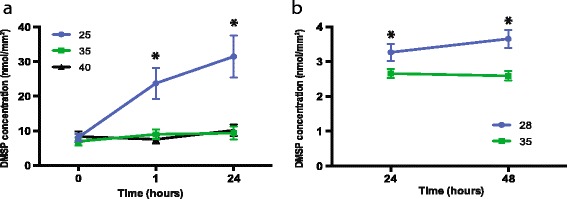
Changes in DMSP concentration (mean ± s.e.) in adult corals (*n = 5*) and settled juveniles (*n = 6*) of the coral *A. millepora*. Adults (**a**) were exposed to ambient/control (35 PSU, *green*) and two salinity stress conditions (25 and 40 PSU in *blue* and *black* respectively). DMSP concentrations increased significantly under hyposaline stress (25 PSU; *H-F Pr < 0.005) and through time compared to both the control and hypersaline stress conditions (40 PSU; *p adj < 0.05). No significant changes in DMSP levels were observed between the control and 40 PSU treatments. Juveniles (**b**) were exposed to ambient/control (35 PSU, *green*) or hyposaline (28 PSU, *blue*) conditions. In this case, DMSP levels differed significantly between treatments and controls (F = 17.70, *p < 0.0005), but did not differ significantly with time

Exposure of adult *A. millepora* colonies to a sudden decrease in salinity (25 PSU) resulted in a 2.6 fold increase in tissue DMSP concentration after 1 h (from 9.02 nmol mm^−2^ at 35 PSU to 23.76 nmol mm^−2^ in the treatment) compared to the controls. DMSP levels in these colonies continued to increase through time, reaching 31.46 nmol mm^−2^ after 24 h, representing a 3.5 fold increase in DMSP relative to the control (TukeyHSD, p adj <0.05; Fig. 2a and Additional file 1: Table S1). In aposymbiotic *A. millepora* juveniles, exposure to low salinity (28 PSU) also triggered an increase of DMSP levels of 1.2 fold after 24 h (from 2.66 nmol mm^−2^ at 35 PSU to 3.27 nmol mm^−2^ in the treatment) and of 1.4 fold after 48 h relative to control juveniles maintained at 35 PSU (ANOVA, *p* < 0.0005; Fig. 2b and Additional file 1: Table S3).

In contrast, adult *A. millepora* nubbins exposed to hypersaline conditions (40 PSU) exhibited no significant change in tissue DMSP concentrations compared to the controls (TukeyHSD, p adj >0.05; Fig. 2 and Additional file 1: Table S1). At both time points, the concentration of the DMSP breakdown product acrylate did not differ significantly from controls in either treatment (Additional file 4: Figure S1). Furthermore, no clear physiological changes were observed in the corals during the 24 h period of both hypo- and hypersalinity stress experiments, as assessed by PAM fluorometry (MANOVA, H-F Pr > 0.05; Additional file 5: Figure S2, Additional file 1: Table S2) and *Symbiodinium* cell density (Additional file 5: Figure S2).

### Differential gene expression and candidate DMSP biosynthesis gene identification

5.5–10.2 million RNAseq reads were obtained for each adult coral sample while 3.4–8.8 million reads were obtained for each juvenile coral sample (GEO reference GSE96916). Principal component analysis (PCA) of the count matrix of the 26,622 *A. millepora* gene predictions revealed that while colony had a strong influence on grouping in the case of the adult samples, the juveniles were clearly separated by treatments (Additional file 6: Figure S3). In both adult corals and juveniles, the number of differentially expressed genes (DEGs; FDR < 0.05) was higher after 24 h compared to 1 h in the adults and 48 h in the juveniles (Additional file 7: Figure S4).

BlastP analysis of the *A. millepora* gene predictions led to the identification of coral members of gene families implicated in DMSP biosynthesis in other organisms (Table 2 and Additional file 2: Table S4), some of which were differentially expressed in response to hyposaline stress and on this basis are considered as candidates for roles in DMSP biosynthesis in corals. Amongst the genes up-regulated under hyposaline conditions were members of each class of enzyme in the DMSP biosynthesis pathway previously described in the alga *Ulva intestinalis* [11], whereas there was no evidence for up-regulation of genes encoding enzyme classes implicated in DMSP-synthesis in higher plants (DMSP-amine oxidase and *S*-methylmethionine (SMM) transaminase-decarboxylase; Table 2 and Fig. 1a and b, step 3).

Six transaminase family members (Table 2, AT1- AT6) were identified as candidates for the initial aminotransferase step in the algal biosynthetic pathway (conversion of L-methionine to 2-oxo-4-methylthiobutanoate; MTOB), on the basis of elevated levels of expression in adults and/or juveniles during hypo-osmotic stress. One of these candidate genes, AT1 was expressed at higher levels at both time points in both juveniles and adults, and is therefore of particular interest. Although BlastP NR database comparisons classified the AT1 predicted protein as most similar to ethanolamine-phosphate phospholyases (EC2.6.1.88), its overall sequence similarity (5E^−35^) to the aminotransferase candidate (269005) from the diatom *Fragilariopsis cylindrus* [12] is consistent with the hypothesis that the two proteins play analogous roles in DMSP metabolism. While the expression levels of five other aminotransferases (AT2 – AT6) were less consistent across the treatments, BlastP NR comparisons imply that their transamination reactions are likely to be 2-oxoglutarate dependant and hence cannot be excluded as candidates for roles in DMSP biosynthesis (Additional file 2: Table S4).

The second step in the algal DMSP biosynthesis pathway involves the reversible reduction of MTOB to 4-methylthio-2-hydroxybutyrate (MTHB), but this reaction is not restricted to DMSP-producing organisms [43]. Table 2 lists the differentially expressed genes (REDOX1-REDOX10) that encode NAD- or NADP-dependant dehydrogenases. Due to their redox capacities, the dehydrogenases corresponding to EC1.2.1.3 (Table 2, REDOX2, REDOX3, REDOX5 and REDOX8) could equally well correspond to the enzyme carrying out the terminal step (oxidation of DMSP-aldehyde; DMS-ald) in the plant DMSP biosynthetic pathway or that which converts MTOB to MTHB in the algal pathway. REDOX1 was consistently up-regulated in adult and juvenile corals with database comparisons indicating that it is a 10-tetrahydrofolate reductase since the N-terminal part of the protein contains a hydrolase domain highly specific for this class of enzyme (5.79E^−144^ similarity with NCBI cd08647). Moreover, TargetP (http://www.cbs.dtu.dk/services/TargetP/) predicts that REDOX1 is mitochondrial, which is consistent with the location of the best NR database matches and therefore of relevance to its ability to function in DMSP synthesis. REDOX2 and REDOX3 were differentially up-regulated in the adults when excluding time as a factor (Additional file 2: Table S5), and significantly up-regulated in juveniles (at 24 h in the case of REDOX3; at both time points for REDOX2). REDOX2 may be the best candidate for enzymatic reduction of MTOB, as it matches (9.31E^−12^) to a dehydrogenase (177646) that is highly up-regulated in the diatom *F. cylindrus* under conditions that lead to DMSP biosynthesis via the algal pathway [12].

Both the plant and algal DMSP biosynthesis pathways feature *S*-adenosylmethionine-dependent (SAM-dependent) methylation steps. In the algal pathway, conversion of MTHB to dimethysulphonio-2-hydroxybutyrate (DMSHB) involves a SAM-dependant methyltransferase, as does the conversion of methionine to SMM in the plant pathway (Fig. 1). Two methyltransferases (METHYL1 and METHYL2) were up-regulated during salinity stress (Table 2), although database comparisons suggest other primary roles for both METHYL1 and METHYL2 due to their methyltransferase domains (NCBI cd02440) being class I type, as is also the case for the methionine *S*-methyltransferase Q9LTB2 (which functions in the plant DMSP pathway), and the algal methyltransferase (212856) identified by Lyon et al. [44]. Of the candidates, METHYL1 was the most consistently up-regulated in the hyposaline treatments. A third SAM-dependant methyltransferase METHYL3 (Table 2), was initially identified as the most likely candidate for the conversion of MTHB to DMSHB [7] based on its similarity to the primary candidate for this role in the alga *F. cylindrus* [12]. Note, however, that METHYL3 was not differentially expressed as a result of exposure to altered salinity conditions.

The final step in the algal DMSP biosynthesis pathway, the transformation of DMSHB to DMSP, is the least well understood. The enzyme involved is thought to be an oxygen dependant decarboxylase [43], but has not been characterised. Four candidate enzymes (DECARB1-DECARB4) were identified in the coral on the basis of similarity with the diatom decarboxylases implicated in DMSP biosynthesis [44], but neither these nor the candidates from the diatom are likely to be oxygen-dependent. All of the four *Acropora* candidates encode pyridoxal phosphate (PP) dependent decarboxylases; like the diatom candidate 263016 [12], DECARB1 encodes a group IV PP-dependent decarboxylase (Pfam02784). The remaining three coral candidate decarboxylases are of the group II PP-dependent type (Pfam00282). None of these coral candidate decarboxylases showed consistent up-regulation across the hyposaline manipulation experiments (Table 2).

### Differential expression of genes involved in methionine metabolism

**Fig. 3:**
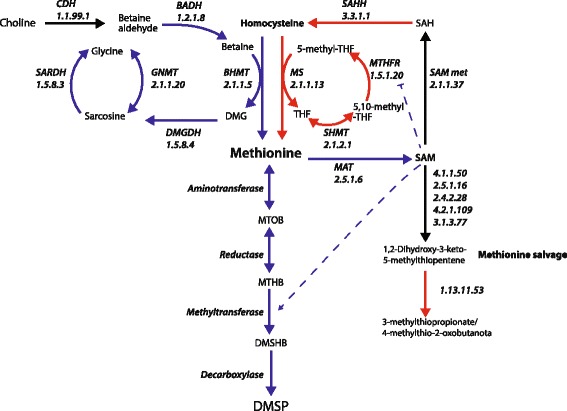
Changes in expression levels of genes involved in methionine metabolism during hyposaline stress in the coral *A. millepora*. Enzyme names and EC numbers are shown in italics (names as in Table 1). *Blue*, *red* or *black arrows* represent steps where genes are up-regulated, down-regulated or do not change significantly, respectively, during hyposaline stress in adult and/or juvenile corals. *Dashed arrows* indicate other roles of SAM (FDR <0.05, see Additional file 2: Table S4, for values). Dimethylglycine (DMG); tetrahydrofolate (THF). Abbreviations for compounds are as in the legend to Fig. 1

Although methionine adenosyltransferases (MAT1 and MAT2), which convert methionine to its activated form (*S*-adenosyl methionine), were up-regulated under hyposaline conditions (Table 2; Fig. 3), other coral genes implicated in methionine salvage and the methyl cycle (Table 2) were down-regulated. Methionine synthase (MS), which methylates homocysteine to regenerate methionine, was down regulated in both adults and juveniles, as were the other methyl cycle enzymes, methylenetetrahydrofolate reductase (MTHFR) and serine hydroxymethyltransferase (SHMT; Table 2). Although methionine synthase was down-regulated under hyposaline conditions, methionine can also be generated by methylation of homocysteine by the action of betaine-homocysteine methyltransferase (BHMT; Fig. 3), two coral homologs of which (BHMT1 and BHMT2) were up-regulated in both adults and juveniles (Additional file 2: Table S4). In addition to generating methionine, the action of BHMT converts betaine to dimethylglycine (DMG), which can be converted to glycine by a series of enzymes (Fig. 3; DMGDH (EC1.5.8.4), SARDH (EC1.5.8.3) and GNMT (EC2.1.1.20), all of which were up-regulated under hyposaline conditions (Additional file 2: Table S4). It is also interesting to note that, of a list of genes potentially involved in methionine salvage from SAM (Fig. 3, EC 4.1.1.50, 2.5.1.16, 2.4.1.28, 4.2.1.109 and 3.1.3.77), the only gene differentially expressed under hyposaline conditions enabling the final conversion to 3-methylthiopropionate of this pathway (Fig. 3, EC1.13.11.53) and was down-regulated (Additional file 2: Table S4) in both adults and juveniles. Finally, the coral homolog to the enzyme involved in the methionine trans-sulphuration pathway (cystathionine γ-lyase (CGL), EC4.4.1.1; Additional file 2: Table S4) was not differentially expressed, providing further evidence that methionine is not shunted into either the methyl cycle or the methionine salvage pathways, but rather being driven into DMSP biosynthesis.

## Discussion

### Corals increase production of DMSP under hyposaline stress

DMSP concentrations in adult corals increased 3.5 fold after 24 h exposure to 25 PSU with similar trends observed for aposymbiotic coral juveniles. Increased DMSP production under hyposaline conditions argues against a role for this compound in osmoregulation in corals and contrasts with the situation in a number of other organisms [45, 46] where DMSP biosynthesis and storage increases under hypersaline conditions. Importantly, in the case of *A. millepora*, DMSP concentrations did not change significantly under hypersaline conditions (40 PSU), indicating that corals might use different mechanisms to adjust to changes in osmotic conditions. Increased levels of DMSP have previously been observed in *Acropora* exposed to heat stress, direct sunlight, and air exposure [17]. Taken together, these results suggest that increases in DMSP concentration in the coral (animal and *Symbiodinium*) might be a more general response to stress, although DMSP levels did not increase when *Montastraea franksi* was exposed to copper stress [47].

DMSP has been shown to function as a scavenger of hydroxyl radicals and reactive oxygen species (ROS) generated under high light and UV stress [48, 49]. The observed increase in DMSP levels under hyposaline conditions is consistent with possible antioxidant functions, but does not follow the expected pattern for an osmolite [50], where DMSP levels would be expected to increase under hypersaline conditions [51–53] and decrease in hyposaline conditions [18]. The sharp increase in DMSP concentrations reported here under hyposaline conditions indicates that DMSP is unlikely to function as an osmolyte in corals.

### Coral enzymes with possible roles in DMSP synthesis via an alga-like pathway

**Fig. 4:**
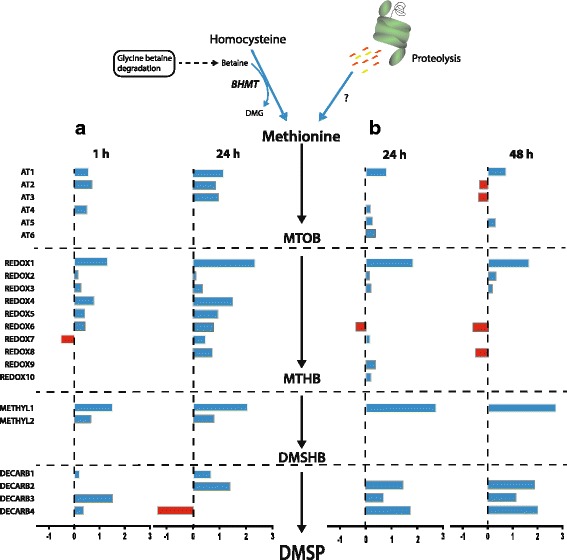
Summary of changes in expression levels of coral genes that are candidates for involvement in an algal-like pathway of DMSP synthesis. For each candidate gene, transcripts levels are indicated as a *bar*, the length of which indicates log_2_-fold change (as in the *x* axis) relative to control in (**a**) adult and (**b**) juvenile corals. *Blue bars* and *red bars* represent the expression levels of up-regulated and down-regulated genes, respectively. Values of candidate gene expression are in Table 2, and abbreviations are as in Fig. 1 and Table 1

RNA sequencing results presented here are consistent with the hypothesis that corals produce DMSP via an alga-like pathway [7], but the identities of genes and enzymes involved needs to be revisited in the light of the transcriptomic responses reported here. Clear differences were observed between adults and juveniles with respect to the responses of genes that are considered candidates for roles in DMSP synthesis by corals (Fig. 4), presumably as consequences of the presence of the dinoflagellate symbionts in the former but not the latter.

In the proposed algal-like pathway of DMSP synthesis, the transamination of methionine and subsequent reduction/oxidation step are both known to be reversible and, while not specific to DMSP producers, exhibit high activity in DMSP accumulating organisms [43]. The gene referred to here as AT1 is considered the best candidate for involvement in the initial transamination step, as it was up-regulated in both adults and juveniles at all time points. In the case of the reduction step, three candidate genes (REDOX1-REDOX3) were up-regulated in all the datasets, whereas the expression data for REDOX8, previously identified as a candidate on the basis of similarity with the diatom reductase [7] were less consistent. Although REDOX1 showed the most consistent up-regulation of expression across the datasets, its likely mitochondrial localisation may limit its involvement in the proposed pathway, hence REDOX2/3 are also considered to be candidates for roles in DMSP production.

The last two steps in the proposed DMSP biosynthesis pathway involve methylation followed by decarboxylation and, unlike the transamination and oxidation/reduction steps, are not reversible. The enzyme referred to here as METHYL3 was initially identified as a candidate for the methylation step [7] on the basis of similarity to a candidate for the same step from a diatom [12] but the corresponding gene was not up-regulated in the present study (Table 2). However, one other putative SAM-dependant methyltransferase (METHYL1) was highly up-regulated across the hyposaline treatment datasets and is thus a candidate for involvement in DMSP biosynthesis.

The identities of genes or enzymes associated with the decarboxylation step of DMSP synthesis are unknown. Two candidates for this role in diatoms have been put forward [12], but neither of these enzymes is likely to be oxygen-dependent, which is inconsistent with earlier metabolic data for this step [54]. No clear candidates for this role emerged from the hyposaline treatment experiments described here.

### Corals do not use a plant-like pathway for DMSP synthesis

Some steps in the algal and higher plant DMSP pathways are biochemically similar, but it is unlikely that the production of DMSP by corals occurs through a plant-like pathway. Possible coral equivalents of S-methyl-L-methionine decarboxylase (SDC) (Table 2, DECARB1), and two DMSP-amine oxidases (Table 2, DOX1 and DOX2) (Fig. 1b, step 3) are present, but the two DOX homologs were down-regulated in both the adults and juveniles in response to hyposaline stress, making their involvement in DMSP production by coral unlikely. The oxidation of DMSP-aldehyde to DMSP (Fig. 1a and b, step 4) in the plant pathway is biochemically similar to the reductase step of the algal pathway (Fig. 1c, step 2 and Fig. 4), hence the observed up-regulation of REDOX candidate genes is the only evidence that the corals could use a plant-like DMSP pathway.

### DMSP production in corals in response to hypo-osmotic stress

The increased production of DMSP in corals under hyposaline stress precludes an osmoregulatory function, but is consistent with a role in conferring protection against ROS generated under these conditions. DMSP is produced in some organisms (e.g. the alga *Tetraselmis subcordiformis*) in response to the availability of excess methionine [55, 56], and this situation may occur in corals in response to hyposaline conditions. Indeed, in *Acropora aspera,* free amino acid (FAA) concentrations have been shown to increase 2.6-fold after 1 h of exposure to hyposaline (28 PSU) conditions [57] but remained unchanged under hypersaline (42 PSU) conditions. Thus, under hyposaline stress, the concentration of free methionine, the precursor of DMSP, is likely to increase in coral tissue.

Osmoregulation has not been extensively studied in corals, but betaines are likely to have major roles as osmolytes. Yancey et al. [58] surveyed a range of osmolyte candidates in seven corals and some other cnidarians, identifying glycine betaine (also known as *N,N,N*-trimethyl glycine) as the dominant osmoregulatory molecule in all of the corals studied except *Porites* species. Similarly, glycine betaine was also implicated as the primary osmolyte in developing larvae of the mushroom coral *Fungia scutaria* [59]. High concentrations of betaines, particularly glycine betaine and taurine betaine, in *Madracis* spp. corals have been confirmed [60]. Increasing levels of betaines correlated with higher light exposure in *Madracis*, suggesting roles in ROS scavenging [60].

*Acropora* spp. produce significant concentrations of glycine-betaines [61] and the responses of these corals to hypo-osmotic stress should be viewed in the context of the requirement to decrease internal osmolarity by reducing betaine levels. Betaines are catabolised via methionine and in the present study, betaine aldehyde dehydrogenase (EC1.2.1.8; BADH) and betaine homocysteine methyltransferase (EC2.1.1.5; BHCMT) were up-regulated in response to hyposaline conditions, which is consistent with betaine breakdown. The action of BHCMT generates methionine and dimethylglycine, the latter of which is metabolised to glycine (and hence to central metabolism) via sarcosine by the sequential actions of dimethylglycine dehydrogenase (EC1.5.8.4; DMGDH) and either glycine-*N*-methyltransferase (EC2.1.1.20; GNMT) or sarcosine dehydrogenase (EC1.5.8.3), all of which were up-regulated under hyposaline conditions in the present study. Because of the flux of homocysteine to methionine driven by betaine catabolism, methionine synthase activity is redundant, which can account for the observed down-regulation of this enzyme (EC2.1.1.13) and the others of the methyl cycle. Some methionine is rescued by conversion to the activated form *S*-adenosyl methionine (note that methionine adenosyltransferase is up-regulated under hyposaline conditions), while the excess is converted to DMSP via the pathways discussed above. Excess DMSP can be metabolised by coral-associated bacteria and *Symbiodinium* into volatile DMS [62, 63], effectively removing it from the system. Note that some homocysteine can be directed into cysteine biosynthesis in other animals (and possibly other corals), however, *Acropora* spp. lack the enzyme cystathionine β-synthase (EC4.2.1.22; [64]).

In addition to being produced as a consequence of betaine catabolism, methionine (and cysteine) will arise in corals as a consequence of proteolysis, which is clearly implied by the up-regulation of many genes encoding proteasome components observed during hypo-osmotic stress (Additional file 2: Table S6).

## Conclusions

Hyposaline stress increased DMSP production in both adults and aposymbiotic juvenile corals, and transcriptomic analyses highlight the potential involvement of specific candidate genes in the production of DMSP via an alga-like pathway. The DMSP produced is likely to provide protection against increased levels of ROS arising as a consequence of stress, but may also constitute a molecular sink for methionine resulting from osmolyte catabolism as well as proteolysis. The biochemistry of DMSP production is not well established for any eukaryotic system and the transcriptomic data presented here have enabled the identification of candidates for roles in DMSP biosynthesis in corals. These results represent an important first step towards understanding the contribution of the coral host to the extremely high DMSP concentrations recorded in coral reefs, and towards a deeper understanding of the cellular functions of this key molecule.

## Additional files

Additional file 1: **Table S1.** Statistical significance tests on DMSP data for adult *A. millepora* under salinity stress: (A) MANOVA, and (B) Tukey post-hoc test. **Table S2.** MANOVA statistical significance test results for PAM data from adult *A. millepora* under salinity stress. **Table S3.** Summary and test statistics for an ANOVA of DMSP concentration in juvenile *A. millepora* under salinity stress. (XLSX 37 kb)

Additional file 2: **Table S4.** *A. millepora* genes differentially expressed in response to hyposaline stress. **Table S5.** *A. millepora* aldehyde dehydrogenases that were differentially expressed in response to hyposaline stress, independent of the time factor. **Table S6.** *A. millepora* proteasome genes differentially expressed in response to hyposaline stress. (XLSX 81 kb)

Additional file 3: Predicted protein sequences from *A. millepora* considered as candidates for roles in DMSP production. (TXT 36 kb)

Additional file 4: Figure S1. Changes in acrylate concentration in tissues of adult corals and settled juvenile *A. millepora* during salinity stress. (PDF 95 kb)

Additional file 5: Figure S2. Density and photosynthetic efficiency of *Symbiodinium* cells within adults of the coral *Acropora millepora* under control and two salinity stress conditions. (PDF 108 kb)

Additional file 6: Figure S3. Principal component analysis (PCA) of normalized gene expression values for individual coral samples in salinity stress experiments. (PDF 101 kb)

Additional file 7: Figure S4. Histograms representing total numbers of differentially expressed genes for each of the salinity stress datasets. (PDF 28 kb)

## Abbreviations

BADH: Betaine aldehyde dehydrogenase
BHMT: Betaine-homocysteine methyltransferase
CDH: Choline dehydrogenase
CGL: Cystathionine γ-lyase
DEG: Differentially expressed gene
DMG: Dimethylglycine
DMGDH: Dimethylglycine dehydrogenase
DMS: Dimethylsulfide
DMSHB: Dimethylsulfonio-2-hydroxybutyrate
DMSO: Dimethylsulfoxide
DMSP: Dimethylsulfoniopropionate
FAA: Free amino acid
FDR: False discovery rate
FSW: Filtered seawater
GNMT: Glycine N-methyltransferase
MAT: Methionine adenosyltransferase
MS: Methionine synthase
MTHB: 4-methylthio-2-hydroxybutyrate
MTHFR: Methylenetetrahydrofolate reductase
MTOB: 2-oxo-4-methylthiobutanoate
PAM: Pulse amplitude modulated
PP: Pyridoxal phosphate
PSU: Practical salinity unit
ROS: Reactive oxygen species
SAH: *S*-adenosylhomocysteine
SAHH: *S*-adenosylhomocysteine hydrolase
SAM: *S*-adenosylmethionine
SARDH: Sarcosine dehydrogenase
SDC: *S*-methyl-L-methionine decarboxylase
SHMT: Serine hydroxymethyltransferase
SMM: *S*-methylmethionine
