# Supplementary figures and images for: Transcriptomic analysis of the response of Acropora millepora to hypo-osmotic stress provides insights into DMSP biosynthesis by corals
Source: BMC Genomics. 2017 Aug 14;18:612. doi: 10.1186/s12864-017-3959-0 (PMC5557254; doi:10.1186/s12864-017-3959-0)

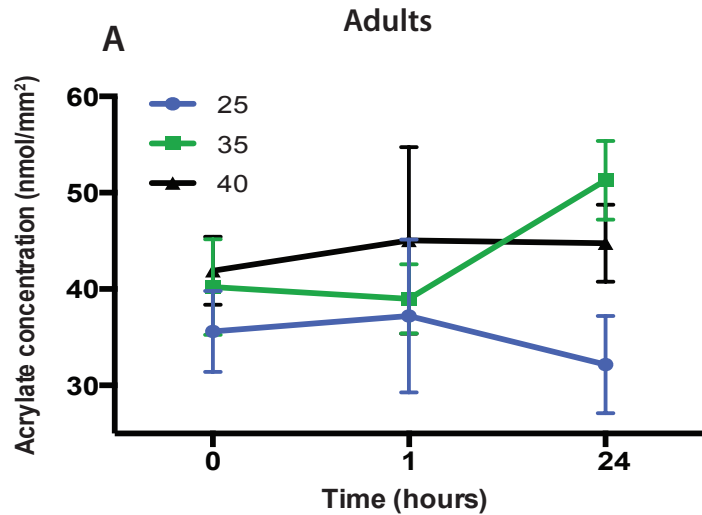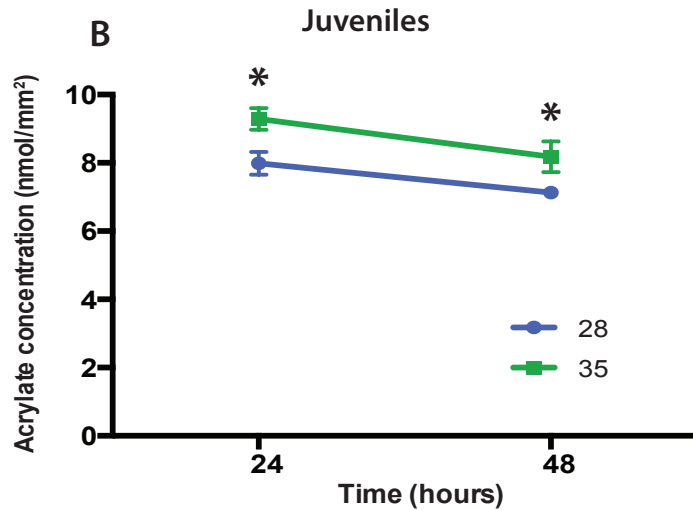

Supplement: Supplementary file 4 — Changes in acrylate concentration in tissues of adult corals and settled juvenile A. millepora during salinity stress. (PDF 95 kb) [file 12864_2017_3959_MOESM4_ESM.pdf]

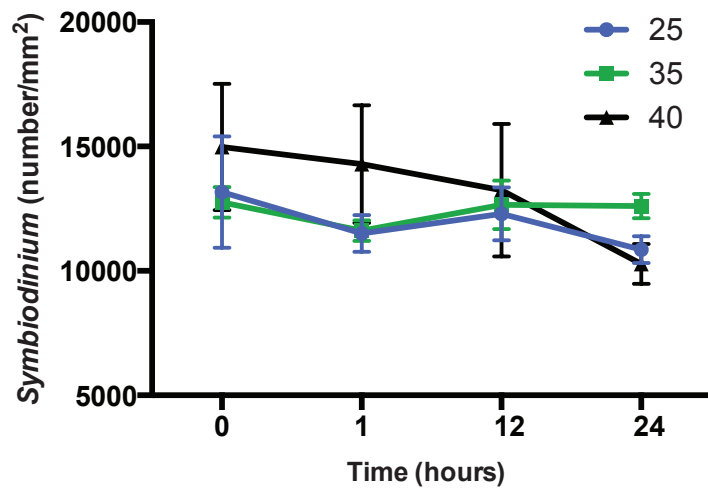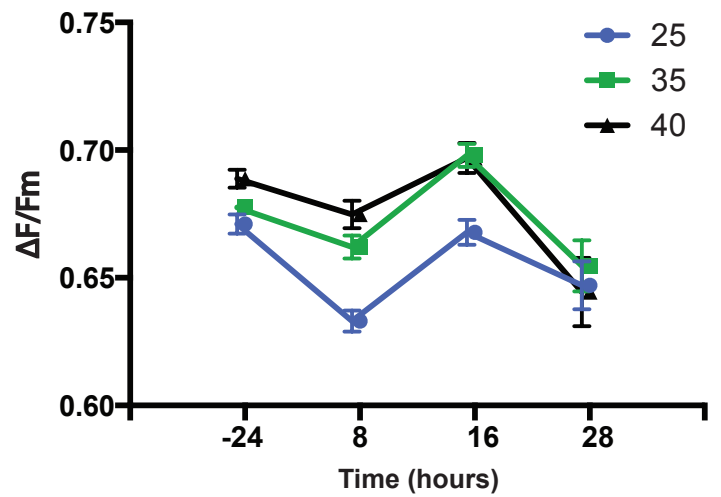

Supplement: Supplementary file 5 — Density and photosynthetic efficiency of Symbiodinium cells within adults of the coral Acropora millepora under control and two salinity stress conditions. (PDF 108 kb) [file 12864_2017_3959_MOESM5_ESM.pdf]

A

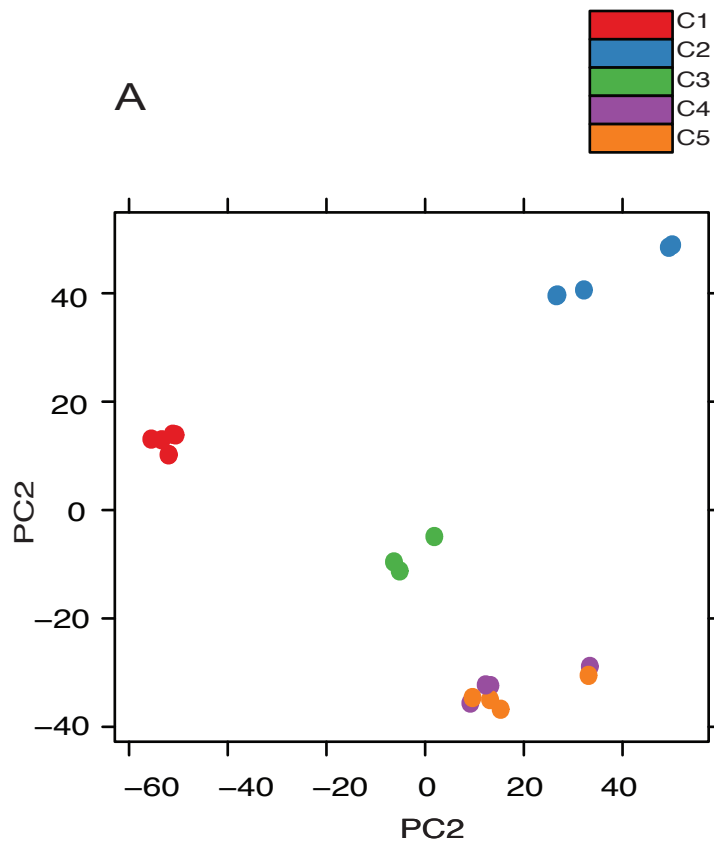

B

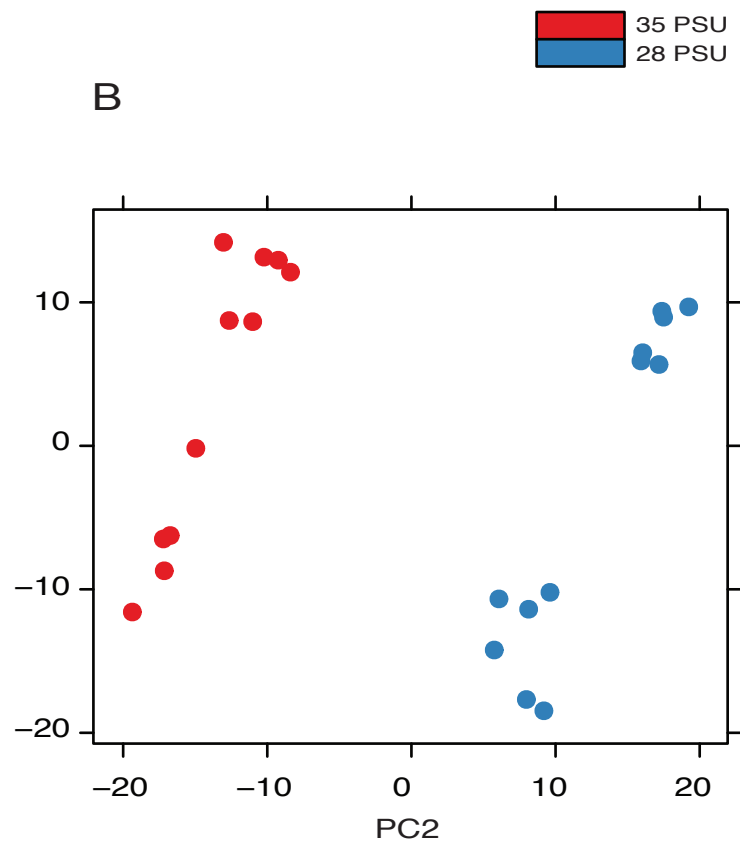

Supplement: Supplementary file 6 — Principal component analysis (PCA) of normalized gene expression values for individual coral samples in salinity stress experiments. (PDF 101 kb) [file 12864_2017_3959_MOESM6_ESM.pdf]

Number of DGs (FDR < 0.01)

3000

2500

2000

1500

1000

500

0

up

down

1 h

24 h

24 h

48 h

Adults

Juveniles

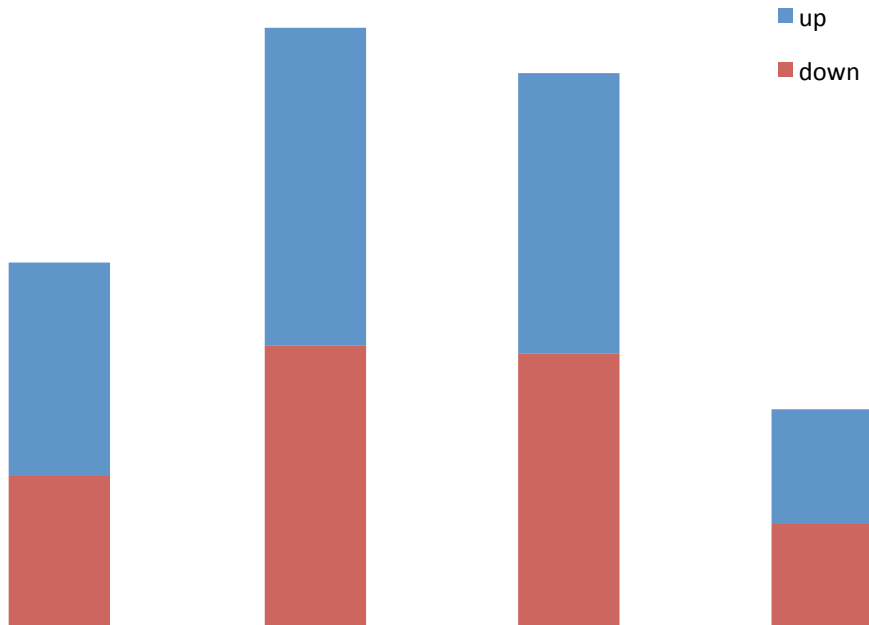

Supplement: Supplementary file 7 — Histograms representing total numbers of differentially expressed genes for each of the salinity stress datasets. (PDF 28 kb) [file 12864_2017_3959_MOESM7_ESM.pdf]
